# Large Language Models for Automating Clinical Trial Criteria Conversion to Observational Medical Outcomes Partnership Common Data Model Queries: Validation and Evaluation Study

**DOI:** 10.2196/71252

**Published:** 2025-10-16

**Authors:** Kye Hwa Lee, Sujung Jang, Grace Juyun Kim, Sukyoung Park, Doeun Kim, Oh Jin Kwon, Jae-Ho Lee, Young-Hak Kim

**Affiliations:** 1 Department of Information Medicine, Department of Digital Medicine, Asan Medical Center, University of Ulsan College of Medicine, 88 Olympic-ro 43-gil, Songpa-gu, Seoul, 05505, Republic of Korea, 82 10-3010-5991, 82 2-3010-2531; 2 Department of Biomedical Engineering, AMIST, Asan Medical Center, University of Ulsan College of Medicine, Seoul, Republic of Korea; 3 Big Data Research Center, Asan Institute for Life Sciences, Asan Medical Center, Seoul, Republic of Korea; 4 Department of Emergency Medicine, Department of Information Medicine, Asan Medical Center, University of Ulsan College of Medicine, Seoul, Republic of Korea; 5 Division of Cardiology, Department of Information Medicine, Asan Medical Center, University of Ulsan College of Medicine, Seoul, Republic of Korea

**Keywords:** clinical trials, eligibility criteria, natural language processing, large language models, hallucination, Observational Medical Outcomes Partnership Common Data Model, OMOP CDM, SQL generation, feasibility assessment

## Abstract

**Background:**

Real-world data–based feasibility assessments enhance clinical trial design, but automating eligibility criteria conversion to database queries is hindered by challenges related to ensuring high accuracy and generating clear, usable outputs.

**Objective:**

The aim of this study is to develop an automated system converting free-text eligibility criteria from ClinicalTrials.gov into Observational Medical Outcomes Partnership Common Data Model (OMOP CDM)–compatible Structured Query Language (SQL) queries and systematically evaluate hallucination patterns across multiple large language models (LLMs) to identify the optimal deployment strategies.

**Methods:**

Our system employs a three-stage preprocessing pipeline (segmentation, filtering, and simplification) achieving 58.2% token reduction while preserving clinical semantics. We compared GPT-4 concept mapping performance against USAGI using 357 clinical terms from 30 trials. For comprehensive evaluation, we analyzed 760 SQL generation attempts (19 trials×8 LLMs×5 prompting strategies) using the SynPUF (Synthetic Public Use Files) dataset and validated selected queries against National COVID Cohort Collaborative reference concept sets using Asan Medical Center’s OMOP CDM database.

**Results:**

GPT-4 achieved a 48.5% concept mapping accuracy versus USAGI’s 32.0% (*P*<.001), with domain-specific performance ranging from 72.7% (drug) to 38.3% (measurement). Surprisingly, the open-source llama3: 8b model achieved the highest effective SQL rate (75.8%) compared to GPT-4 (45.3%), attributed to lower hallucination rates (21.1% vs 33.7%). The overall hallucination rate was 32.7%, with wrong domain assignments (34.2%) and placeholder insertions (28.7%) being the most common. Clinical validation revealed mixed performance: high concordance for type 1 diabetes (Jaccard=0.81), complete failure for pregnancy (Jaccard=0.00), and minimal overlap for type 2 diabetes (Jaccard=0.03), despite perfect overlap coefficients in both diabetes cases. Moderate performance was observed for uncontrolled hypertension (Jaccard=0.18).

**Conclusions:**

While LLMs can accelerate eligibility criteria transformation, hallucination rates of 21‐50% necessitate careful model selection and validation strategies. Our findings challenge assumptions about model superiority, demonstrating that smaller, cost-effective models can outperform larger commercial alternatives. Future work should focus on hybrid approaches combining LLM capabilities with rule-based methods for handling complex clinical concepts.

## Introduction

Clinical trials are essential for medical advancement and drug development, yet they face significant challenges in participant recruitment, with studies showing that 50% of trials fail to meet projected recruitment progress and one-third are terminated due to insufficient recruitment [1-3]. Moreover, only approximately 20% of trials successfully recruit the required participants within the planned timeline [4]. These issues result in delays in new drug approvals and increased research costs, ultimately hindering the development of new therapeutic options [5,6]. To address these challenges, researchers and the pharmaceutical industry are increasingly adopting feasibility assessments and simulations to enhance trial efficiency and success rates [7].

Feasibility assessments are crucial in evaluating the practical, regulatory, and operational aspects of a clinical trial, minimizing risks and setting the foundation for success [8]. By incorporating real-world data (RWD) into the trial design phase, researchers can make more informed decisions, potentially increasing the probability of trial success and reducing the time and costs associated with bringing new therapies to market [9-11]. Leveraging RWD for real-time feasibility assessments during the clinical trial design phase is an effective method to significantly reduce the likelihood of research failure and save time and costs [12,13]. However, the process of transforming clinical trial inclusion or exclusion criteria, which are mostly written in free-text format, into a form suitable for RWD analysis presents several challenges [13,14]. In particular, converting complex clinical concepts, temporal relationships, and elements requiring medical judgment into structured data formats demands considerable expertise and time.

To address these challenges, researchers have been exploring methods to directly query RWD using clinical trial criteria. One notable study by Liu et al developed a system called the Trial Pathfinder, which uses machine learning to evaluate and optimize eligibility criteria for oncology trials using RWD [15]. This approach not only identified opportunities to relax overly restrictive criteria but also demonstrated the potential to increase trial participation by 107% on average while maintaining or improving trial outcomes. However, despite these advances, the automated generation of structured queries from clinical trial criteria faces a critical reliability challenge: Large Language Models (LLMs) frequently generate nonexistent medical concept identifiers—a phenomenon known as hallucination—which can severely compromise query accuracy and patient safety [16].

Recent studies have shown that LLMs, while powerful for natural language understanding, exhibit hallucination rates of 15%‐55% when mapping clinical concepts to standardized vocabularies [17]. This reliability gap poses a significant barrier to clinical deployment, as incorrect concept mappings could lead to missed eligible patients or, worse, inclusion of ineligible participants. Furthermore, the choice of LLM and prompting strategy significantly impacts both performance and cost, yet systematic comparisons across models remain limited. Understanding these trade-offs is essential for developing practical, deployable systems that balance accuracy, efficiency, and economic feasibility.

This study addresses these challenges through a two-pronged approach. First, we present an end-to-end automated system that transforms free-text clinical trial eligibility criteria into Observational Medical Outcomes Partnership Common Data Model (OMOP CDM)-compatible SQL (Structured Query Language) queries using GPT-4 [18], validated with real patient data from Asan Medical Center. Second, we conduct a comprehensive evaluation of hallucination patterns across 8 LLMs (both cloud-based and local) using the Synthetic Public Use Files (SynPUF) [19] dataset, systematically analyzing how model selection and prompt engineering affect reliability. Our findings reveal that while LLMs can accelerate query generation from hours to minutes, hallucination rates of 21%‐50% highlight the need for careful model selection and validation strategies. Surprisingly, our evaluation demonstrated that model size does not necessarily correlate with performance, with smaller open-source models sometimes outperforming larger commercial alternatives in terms of effective SQL generation. By quantifying these trade-offs and identifying optimal model-prompt combinations for different clinical scenarios, this work advances the development of trustworthy and cost-effective AI systems for clinical trial optimization.

## Methods

### Study Design

We conducted a two-phase investigation to develop and validate an automated system for transforming clinical trial eligibility criteria into OMOP CDM [20] SQL queries, followed by systematic analysis of LLM hallucination patterns. The integrated workflow enables both functional validation and reliability assessment of LLM-generated queries in real-world clinical scenarios.

### Data Sources and Study Population

#### Clinical Trials Dataset

We extracted eligibility criteria from the aggregate analysis of ClinicalTrials.gov (AACT) [21] database (accessed July 18, 2023). We utilized two distinct datasets: (1) Development Dataset and (2) Validation Dataset.

First, in the Development Dataset, 30 trials (10 each from breast cancer, diabetes mellitus, and cardiovascular disease domains) were used exclusively for concept dictionary construction and prompt optimization. From these trials, we extracted 357 unique clinical terms for comparative analysis of concept mapping approaches between GPT-4 and USAGI [22].

Second, in the Validation Dataset, 7 high-impact trials were selected based on citation frequency in major medical journals (BRIDGE [Bridging Anticoagulation in Patients who Require Temporary Interruption of Warfarin Therapy for an Elective Invasive Procedure or Surgery; NCT00786474], PARADIGM-HF [Prospective Comparison of ARNI with ACEI to Determine Impact on Global Mortality and Morbidity in Heart Failure; NCT01035255], LEADER [Liraglutide Effect and Action in Diabetes: Evaluation of Cardiovascular Outcome Results; NCT01179048], CHOIR [Correction of Hemoglobin and Outcomes in Renal Insufficiency; NCT00211120], ACT [Randomized Acetylcysteine for Contrast-induced Nephropathy Trial; NCT00736866], DESTINY-Breast04 [Trastuzumab Deruxtecan in Previously Treated HER2-Low Advanced Breast Cancer; NCT03734029], RECOVERY [Randomized Evaluation of COVID-19 Therapy; NCT04381936]) for comprehensive system evaluation.

#### Clinical Data Infrastructure

Real-world validation utilized OMOP CDM version 5.3 data from Asan Medical Center, Seoul, South Korea. This comprehensive dataset contains 4,951,000 unique patients with clinical records spanning from May 1989 to December 2020. The study protocol received approval from the Asan Medical Center Institutional Review Board (IRB No. 2024‐0377).

#### Synthetic Data for Hallucination Analysis

To systematically evaluate hallucination patterns, we employed the SynPUF dataset version DE_1_0_1, containing 2.3 million synthetic Medicare beneficiaries with claims data from 2008 to 2010. This dataset’s limited concept coverage (~27,000 of >2 million OMOP concepts) provided an ideal test bed for identifying hallucination behaviors when models encounter concept mapping challenges.

### Clinical Criteria to SQL Transformation Pipeline

**Figure 1.**
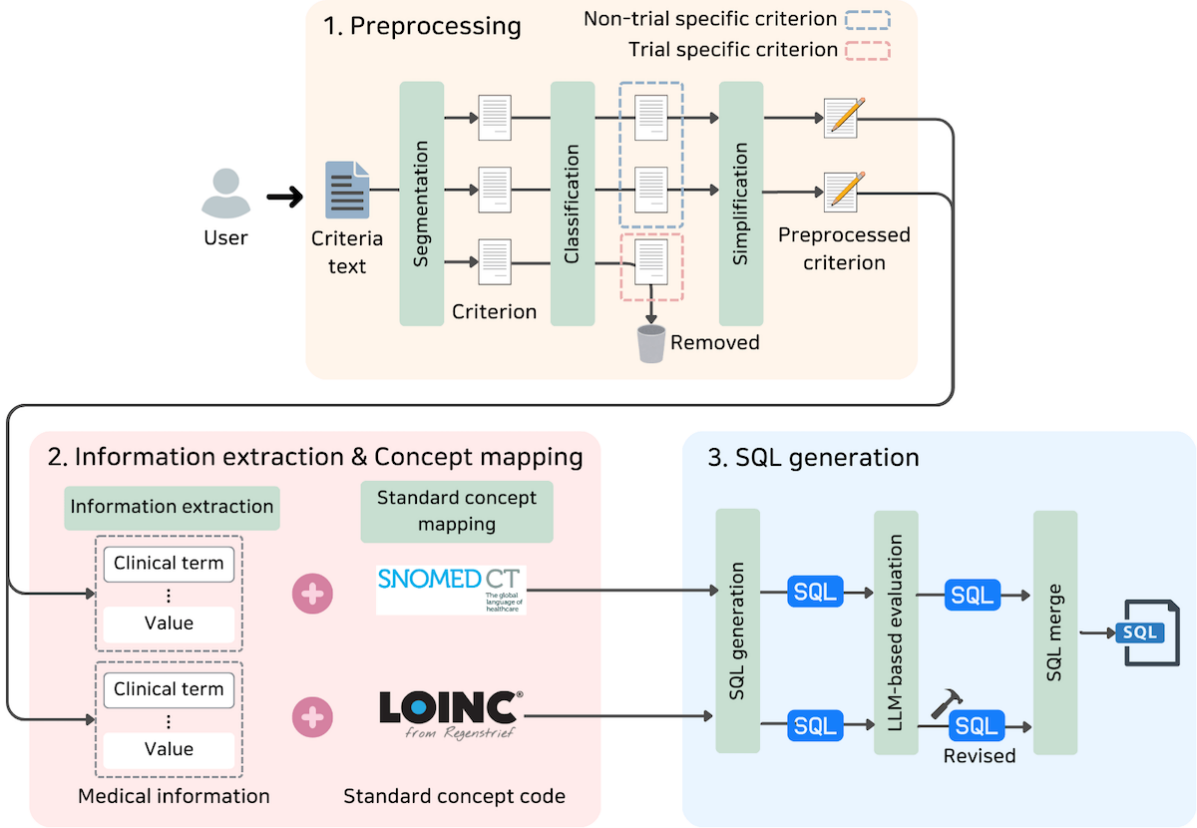
Detailed three-stage preprocessing and concept mapping architecture. Three-stage preprocessing and concept mapping workflow for transforming clinical trial eligibility criteria into Observational Medical Outcomes Partnership Common Data Model (OMOP CDM)–compatible Structured Query Language (SQL) queries.

We developed an automated pipeline that transforms free-text eligibility criteria into OMOP CDM–compliant SQL queries through three interconnected modules. The preprocessing module implements a three-stage approach: (1) segmentation to extract individual criteria while preserving Boolean logic and hierarchical structures, (2) filtration to remove non-queryable trial-specific criteria such as informed consent requirements, and (3) simplification to standardize temporal expressions and reduce token count while maintaining clinical semantics. The information extraction module identifies seven structured elements from preprocessed text, Clinical Terms, Medical Terminology Systems (SNOMED CT, ICD-10, RxNorm, LOINC), Codes, Values, Attributes, Temporal, and Negation, and then maps each clinical term to OMOP-standardized vocabularies using GPT-4. The SQL generation module creates CDM-compliant queries through iterative refinement and optimization. Each module exchanges data via structured JSON to ensure interoperability throughout the pipeline. The preprocessing stages are illustrated in Figure 1, and the detailed architecture of the automated transformation pipeline is shown in Figure S1 of Multimedia Appendix 1. In addition, a comprehensive description of the processing procedures implemented in the automated system is provided in Multimedia Appendix 1.

### Large Language Model Configuration

We employed GPT-4 (March 2024 release) via API with task-specific prompting strategies optimized through iterative refinement. Zero-shot prompting was utilized for straightforward tasks including text segmentation and criteria classification, which minimized token usage and API cost. Few-shot prompting with carefully selected examples enhanced performance for complex tasks including concept mapping and SQL generation. All prompts incorporated explicit instructions for OMOP CDM v5.3 compliance and error handling. Complete prompt templates are provided in Table S1 in Multimedia Appendix 2.

### Comparative Evaluation Framework

#### Clinical Term Mapping Assessment

To evaluate the performance of GPT-4 in mapping clinical terms to standardized concepts, we utilized a previously constructed development dataset comprising 30 clinical trials across three disease domains: breast cancer, diabetes mellitus, and cardiovascular disease. This dataset included diverse expressions of eligibility criteria and clinical terminologies, making it suitable for assessing concept mapping accuracy within the OMOP CDM. We compared GPT-4 with USAGI, an open-source tool developed by the OHDSI community. While GPT-4 is an LLM with advanced natural language understanding capabilities, USAGI uses string normalization, Levenshtein distance–based similarity scoring, and ontology-based heuristics to identify candidate OMOP-standard concepts. In our experiments, USAGI was run using its default settings, and the top-ranked concept based on similarity score was selected for each input term. Relevant clinical terms were extracted from the eligibility criteria of each trial and mapped using both GPT-4 and USAGI. Two clinical experts then jointly reviewed the mapping results from both systems and selected the most appropriate OMOP-standard concept through mutual consensus. In each case, they either chose the better of the two candidate concepts or, when necessary, manually assigned a more suitable concept. This consensus-based evaluation approach reflects real-world clinical decision-making and contributes to the reliability and validity of the reference standard.

#### SQL Query Validation

To rigorously evaluate the accuracy and applicability of the generated SQL queries within the OMOP CDM, we conducted an expert-based validation involving two experts with strong proficiency in both clinical concepts and the OMOP CDM. Generated SQL queries underwent systematic evaluation using 80 predefined evaluation criteria designed to assess three key dimensions: SQL syntax adherence, CDM schema compliance, and criteria contextual accuracy. Each expert independently rated all applicable criteria on a 4-point scale (1=noncompliant and 4=fully compliant), with final scores calculated as the average of both ratings.

The evaluation framework was applied differentially based on query content. For all generated queries, the full set of 80 criteria was used to assess structural and schema-level accuracy. However, for queries containing clinical concepts from our prevalidated concept map, only 21 criteria from the original 80 were applicable—these specifically evaluated concept inclusion accuracy and concept ID correctness. Inter-rater reliability was measured using Cohen’s kappa, with discrepancies resolved through consensus discussion.

This expert-driven evaluation allowed for the identification of critical issues—such as misinterpretation of clinical logic, inaccurate temporal constraints, or improper use of OMOP tables—that automated methods might overlook. As a result, we verified that the generated SQL queries were not only technically correct but also clinically meaningful and suitable for use in real-world, standardized health care data environments. The complete list of evaluation criteria is provided in Table S1 in Multimedia Appendix 3.

#### Clinical Cohort Validation

To assess real-world performance of the automatically generated SQL queries, we conducted cohort extraction experiments using the OMOP CDM dataset from Asan Medical Center. Given the resource-intensive nature of building complete gold-standard concept sets manually, we utilized officially validated OMOP CDM-based concept sets provided by the National COVID Cohort Collaborative (N3C) as reference standards. A total of three clinical trials were included in this evaluation—NCT00211120, NCT00786474, and NCT01179048—which collectively comprised 40 eligibility criteria. These trials were selected because validated N3C concept sets were available for at least one of their eligibility criteria, allowing for meaningful comparison between system-generated cohorts and external reference standards. Each criterion was screened based on two conditions: (1) convertible to OMOP CDM format and (2) availability of validated concept sets from N3C. For these selected criteria—including Pregnancy (NCT00786474), Type 2 diabetes and Type 1 diabetes (NCT01179048), and Presence of uncontrolled hypertension (NCT00211120)—SQL queries were automatically generated using our system and executed against the OMOP CDM database.

 Cohort similarity between system-generated and reference standard cohorts was quantified using two complementary set–based metrics: Jaccard index (intersection/union) and overlap coefficient (intersection/minimum set size). The Jaccard index provides a symmetric measure of overall similarity, while the overlap coefficient indicates the extent to which the smaller cohort is contained within the larger cohort, useful for identifying cases of incomplete concept coverage. This approach enabled objective assessment of query accuracy without requiring manual chart review, leveraging established reference standards for scalable validation. Additionally, to demonstrate technical feasibility, successfully generated SQL queries were executed against the SynPUF dataset to assess cohort retrieval capabilities. Patient counts were recorded for selected trials where queries were executed without errors.

#### Experimental Design

Although GPT-4 was used for initial system development due to its strong performance, the limited concept coverage in SynPUF provided an opportunity to systematically compare hallucination patterns across multiple LLMs, informing optimal model selection for different clinical scenarios. The 19 trials for the main study included breast cancer (6 trials), cardiovascular disease (4 trials), diabetes (3 trials), chronic obstructive pulmonary disease (3 trials), and others (3 trials), with complexity levels categorized as simple (8), moderate (7), and complex (4). We employed a factorial design testing eight LLMs (three cloud-based: GPT-4, GPT-3.5-turbo [23], and Claude-3-sonnet [24]; five locally deployed: Llama3:8b [25], DeepSeek-R1:8b [26], Qwen2.5 [27], Phi3 [28], and Gemma3:4b [29]) with five prompting strategies (zero_shot, structured_approach, explicit_uncertainty, validation_focused, and error_aware). The five prompting strategies were designed to test different approaches to query generation:

- zero_shot: Direct query without examples or guidance
- structured_approach: Step-by-step decomposition guidance
- explicit_uncertainty: Encouraging placeholder use for uncertain mappings
- validation_focused: Emphasizing validation and accuracy requirements
- error_aware: Including SynPUF limitations and common pitfalls

Following a pilot phase (5 trials×8 models×5 prompts=200 queries) for methodology validation, the main study analyzed 760 queries (19 trials×8 models×5 prompts).

#### Hallucination Detection and Classification

An automated detection system was developed to identify hallucinations in generated SQL queries. The system validated concept identifiers against the SynPUF concept inventory and verified domain appropriateness according to OMOP CDM specifications. For the initial validation phase, we analyzed SQL generation failures from seven validation trials. For the large-scale model comparison, we developed a five-category hallucination classification schema ordered by severity. Category A (Critical) comprised nonexistent concept IDs that invalidate query results. Category B (Major) included valid concepts assigned to incorrect domains, violating CDM structural integrity. Category C (Major) captured natural language in concept fields, reflecting confusion between labels and identifiers. Category D (Moderate) consisted of placeholder values requiring manual intervention. Category E (Minor) encompassed easily correctable syntax or schema errors. This classification specifically evaluated model tendencies toward generating inappropriate concept references, distinct from the initial error analysis.

#### Analytical Framework of Model Performance

Model performance was evaluated across four dimensions. The model performance dimension compared effective SQL generation rates, hallucination frequencies, and error distributions across eight LLMs. The prompt strategy dimension assessed five prompting approaches to identify optimal error minimization strategies. The clinical domain dimension analyzed performance variations across condition, drug, measurement, procedure, and observation concepts. The complexity dimension examined correlations between query complexity (criteria count, logical operators, temporal constraints) and error occurrence.

### Statistical Analysis

Statistical analyses were performed using Python 3.11 (Python Software Foundation) with scipy library (version 1.11.4) [30] and statsmodels library (version 0.14.0) [31] libraries. For concept mapping comparisons between GPT-4 and USAGI, McNemar’s test was applied to paired binary outcomes. Three primary performance metrics were calculated: (1) SQL generation rate as the proportion of trials producing syntactically valid SQL, (2) hallucination rate as the proportion of generated queries containing invalid concept IDs, and (3) effective SQL generation rate, calculated as SQL generation rate×(1–hallucination rate), representing queries both syntactically valid and free from concept hallucinations.

Model performance across the eight LLMs was compared using one-way ANOVA followed by Tukey’s honestly significant difference (HSD) test for post hoc pairwise comparisons. Effect sizes were quantified using Cohen *d* to assess the practical significance of differences between prompting strategies. *χ*^2^ tests evaluated the distribution of hallucination types across models. Multiple linear regression analysis identified predictors of hallucination rates, with model type, prompt strategy, and query complexity as independent variables; model fit was assessed using *R*². All statistical tests employed *α*=.05 with Bonferroni correction for multiple comparisons where applicable.

### Ethical Considerations

This study protocol was reviewed and approved by the Institutional Review Board of Asan Medical Center (IRB No. 2024-0377). As this study did not involve human participants, informed consent was not applicable. All data used in the study were de-identified prior to analysis, and no individual-level identifiable information was accessed.

### Data and Code Availability

Source code, including the complete pipeline implementation and hallucination detection system, is available at [https://github.com/sujeong-jang/ctos]. The SynPUF dataset is publicly accessible through Centers for Medicare & Medicaid Services. Clinical trial criteria and evaluation datasets are available upon request with appropriate data use agreements. Detailed documentation for system deployment and reproduction is provided in the repository.

## Results

### Preprocessing and Clinical Concept Extraction

**Figure 2.**
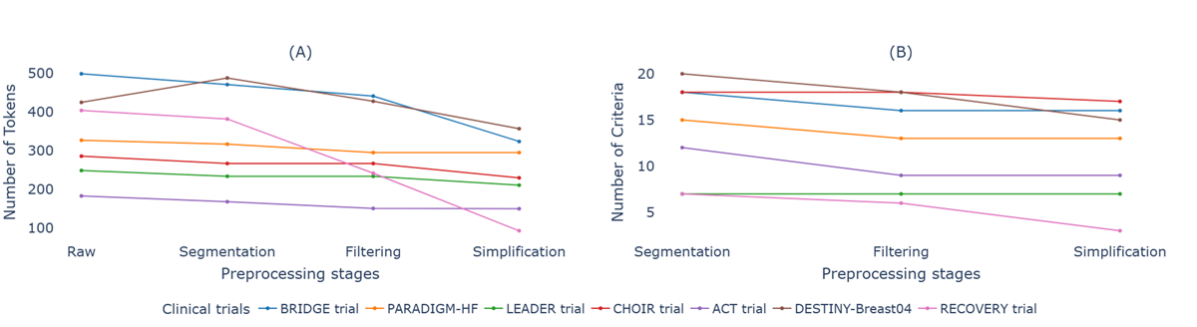
Progressive reduction in token count and criteria through preprocessing stages. (A) Token count changes across segmentation, filtering, and simplification stages for seven validation trials. (B) Corresponding changes in criteria count. Trials shown are as follows: BRIDGE (Bridging Anticoagulation in Patients who Require Temporary Interruption of Warfarin Therapy for an Elective Invasive Procedure or Surgery; NCT00786474), PARADIGM-HF (Prospective Comparison of ARNI with ACEI to Determine Impact on Global Mortality and Morbidity in Heart Failure; NCT01035255), LEADER (Liraglutide Effect and Action in Diabetes: Evaluation of Cardiovascular Outcome Results; NCT01179048), CHOIR (Correction of Hemoglobin and Outcomes in Renal Insufficiency; NCT00211120), ACT (Randomized Acetylcysteine for Contrast-induced Nephropathy Trial; NCT00736866), DESTINY-Breast04 (Trastuzumab Deruxtecan in Previously Treated HER2-Low Advanced Breast Cancer; NCT03734029), RECOVERY (Randomized Evaluation of COVID-19 Therapy; NCT04381936)

Analysis of seven validation trials revealed substantial heterogeneity in eligibility criteria complexity, ranging from 6 to 25 criteria per trial (14.7±6.3). The three-stage preprocessing pipeline systematically reduced linguistic complexity while preserving clinical semantics (Figure 2). Initial segmentation achieved modest token reduction (331.4±85.2) while maintaining structural integrity. Subsequent filtering of non-OMOP–compatible criteria reduced both criteria count (11.71±4.9) and token count (311.6±78.3). Final simplification yielded 10.71±4.2 criteria with 138.57±42.1 tokens per trial, representing a 58.2% overall token reduction.

This preprocessing identified 80 clinically relevant criteria suitable for automated query generation. Information extraction from these 80 preprocessed criteria yielded 188 unique clinical terms successfully mapped to OMOP-compatible elements. Domain distribution analysis revealed that Condition concepts comprised the predominant category (n=93, 49.5%), followed by Drug (n=29, 15.4%), Procedure (n=24, 12.8%), Measurement (n=18, 9.6%), Observation (n=14, 7.4%), Demographic (n=8, 4.3%), Visit (n=1, 0.5%), and Device (n=1, 0.5%) domains (Table 1). Term frequency analysis identified demographic criteria as the most prevalent. “Age” appeared in 6 trials (out of 7 trials, 85.7%; 6 mentions). Clinical terms included “international normalized ratio” (2/7 trials, 28.6%; 2 mentions), “diabetes mellitus” (2/7 trials, 28.6%; 2 mentions), “breast cancer” (1/7 trial, 14.3%; 8 mentions), “pneumonitis” (1/7 trial, 14.3%; 3 mentions), “interstitial lung disease” (1/7 trial, 14.3%; 3 mentions), “transient ischemic attack” (1/7 trial, 14.3%; 2 mentions), and “systemic embolism” (1/7 trial, 14.3%; 2 mentions), as shown in Table 2. Percentages are calculated based on the number of trials in which the term appeared, and mentions indicate the number of criteria within trials in which the term appeared.

**Table 1.** Distribution of extracted clinical concepts by OMOP CDM^a^ domain.

Domains	Count (n=188), n (%)
Condition	93 (49.5)
Drug	29 (15.4)
Procedure	24 (12.8)
Measurement	18 (9.6)
Observation	14 (7.4)
Demographic	8 (4.3)
Visit	1 (0.5)
Device	1 (0.5)

a OMOP CDM: Observational Medical Outcomes Partnership Common Data Model.

**Table 2.** Ten most frequently occurring clinical terms across all trials.

Clinical terms	Trials (n=7), n (%)	Mentions (criterion), n
Age	6 (85.7)	8
International normalized ratio	2 (28.6)	6
Diabetes mellitus	1 (14.3)	3
Breast cancer	1 (14.3)	3
Pneumonitis	1 (14.3)	2
Interstitial lung disease	1 (14.3)	2
Transient ischemic attack	1 (14.3)	2
Systemic embolism	1 (14.3)	2

### Comparative Performance of Concept Mapping Approaches

Evaluation of concept mapping accuracy demonstrated GPT-4’s superior performance compared to USAGI across 357 clinical terms extracted from the 30 development trials. GPT-4 achieved an overall accuracy of 48.5% (173/357) versus USAGI’s 32.0% (114/357), a statistically significant difference (*P*<.001, McNemar’s test). Domain-stratified analysis revealed GPT-4’s highest accuracy in the Drug domain (72.7%, 16/22) and lowest in the Measurement domain (38.3%, 31/81), suggesting particular challenges with laboratory-related concepts requiring numeric threshold interpretation.

Notably, among the 243 terms that USAGI misclassified, GPT-4 correctly mapped 61 (25.1%), demonstrating superior contextual understanding. For instance, USAGI incorrectly mapped “human immunodeficiency virus (HIV)” to “Human immunodeficiency virus contact” (a social context concept), while GPT-4 correctly identified “Human immunodeficiency virus infection” (a clinical diagnosis). This pattern of improved semantic interpretation was consistent across complex multiword medical terms.

### SQL Query Generation Quality and Expert Validation

Two domain experts independently evaluated SQL queries generated from the seven validation trials using 80 predefined criteria across three dimensions: SQL syntax adherence, CDM schema compliance, and criteria contextual accuracy (inter-rater reliability: Cohen κ=0.85). Each criterion was rated on a 4-point scale (1=noncompliant and 4=fully compliant). SQL syntax adherence achieved near-perfect scores from expert reviewers (3.99±0.12) and matched LLM self-evaluation (4.00±0.00), confirming robust syntactic generation capabilities. CDM schema compliance similarly demonstrated strong performance (expert: 3.89±0.21, LLM: 4.00±0.00), indicating successful adaptation to OMOP table structures and relationships.

However, criteria contextual accuracy exhibited greater variability and lower absolute scores (expert: 3.19±0.45, LLM: 3.53±0.32), with the 0.34-point difference suggesting systematic LLM overconfidence in semantic interpretation (*P*=.023, paired *t*-test). This divergence was most pronounced for complex eligibility criteria involving temporal relationships or multicondition logic.

Among the total queries evaluated, 21 contained prevalidated concepts from our development dataset, enabling additional validation of concept mapping accuracy. For these queries, concept inclusion accuracy averaged 3.43±0.38 (expert) versus 3.54±0.29 (LLM), while concept ID correctness achieved higher scores of 3.79±0.24 (expert) versus perfect score of 4.00±0.00 (LLM). The perfect LLM score for concept ID correctness reflects its consistent ability to generate syntactically valid concept identifiers, though not necessarily clinically appropriate ones.

### Clinical Cohort Retrieval Performance

Validation using N3C-provided reference concept sets [32] revealed that performance strongly correlated with criteria complexity (Table 3). For the two evaluated trials (NCT00211120, NCT00786474, and NCT01179048), we assessed four distinct eligibility criteria that had available N3C reference standards. The query for Type 1 diabetes (NCT01179048) demonstrated high retrieval accuracy, achieving a Jaccard index of 0.81 and a perfect overlap coefficient of 1.0, indicating that the system-generated cohort closely matched the reference set. In contrast, Type 2 diabetes showed a very low Jaccard index of 0.03 despite an overlap coefficient of 1.0, suggesting that while the retrieved patients were all included in the reference cohort, a large portion of the reference patients was not captured—likely due to incomplete descendant concept inclusion. The Pregnancy criterion (NCT00786474) resulted in complete retrieval failure, with both the Jaccard index and overlap coefficient equal to 0, indicating that no matching patients were retrieved. This may reflect the system’s inability to handle concept granularity or contextual nuances related to reproductive health data in the OMOP CDM schema. The presence of uncontrolled hypertension criterion (NCT00211120) achieved moderate performance, with a Jaccard index of 0.18 and an overlap coefficient of 0.48, reflecting partial concept mapping success for a chronic condition represented by a mix of clinical measurements and diagnostic codes.

**Table 3.** Comparison of patient cohort retrieval between system-generated queries and reference concept sets.

NCT ID	Simplified criterion	Jaccard similarity	Overlap coefficient
NCT00786474	Pregnancy	0	0
NCT01179048	Type 2 diabetes	0.03	1.0
	Type 1 diabetes	0.81	1.0
NCT00211120	Presence of uncontrolled hypertension	0.18	0.48

These results collectively highlight that the system is capable of achieving high performance when querying well-defined, hierarchically stable clinical concepts (eg, Type 1 diabetes) but may underperform when concept definitions are broad, heterogeneous, or require deep descendant inclusion (eg, Type 2 diabetes or pregnancy). Improvements in concept hierarchy coverage and semantic normalization may be necessary to enhance retrieval for more complex clinical criteria.

### Large-Scale Model Comparison and Hallucination Analysis

**Figure 3.**
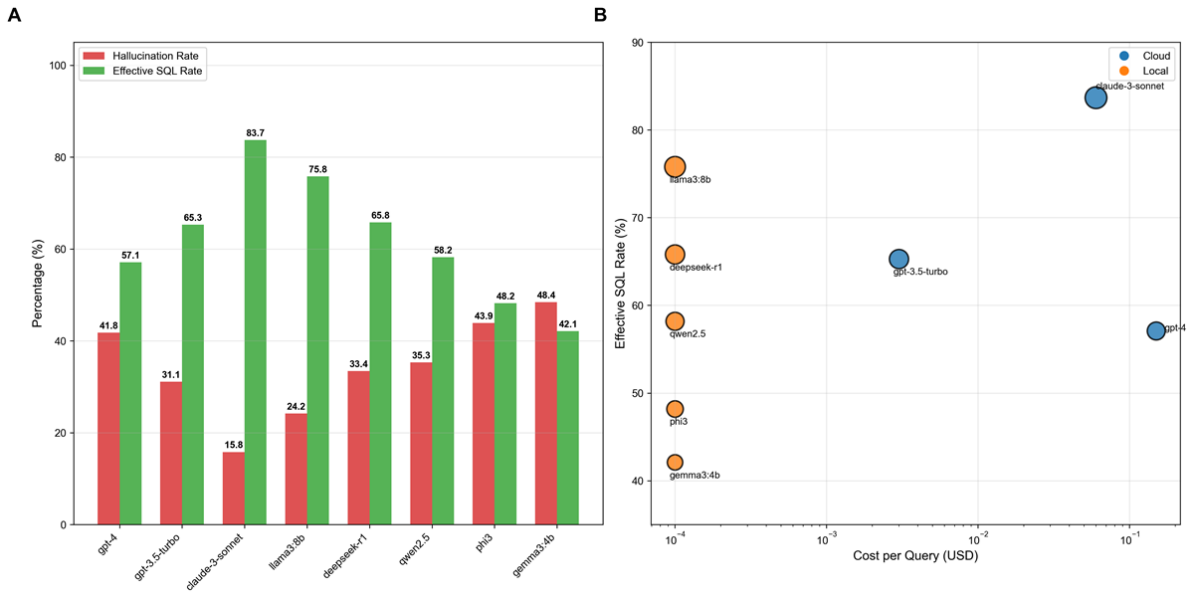
Comprehensive performance analysis of eight large language models for OMOP CDM SQL generation. (A) Comparison of hallucination rates and effective SQL rates across models. (B) Cost-performance trade-off analysis showing the relationship between cost per query (log scale) and effective SQL rate, with bubble size proportional to effective rate. Cloud models (blue) and local models (orange) show distinct clustering patterns. OMOP CDM: Observational Medical Outcomes Partnership Common Data Model; SQL: Structured Query Language.

To systematically evaluate hallucination patterns across multiple LLMs, we conducted a separate large-scale experiment using the SynPUF dataset, analyzing 760 SQL generation attempts (19 clinical trials×8 models×5 prompting strategies), distinct from our initial validation study. Analysis of 760 SQL generation attempts revealed significant heterogeneity in model performance (*F*(7752)=3.36, *P*=.0085), with hallucination rates ranging from 21.1% (n=160) to 49.5% (n=376) among models achieving >80% SQL generation success. The overall hallucination rate was 32.7% (n=249) (95% CI 29.4%‐36.1%), with substantial variation in both generation capability and accuracy across models (Figure 3 and Table 4).

**Table 4.** Model performance summary.

Model	Architecture	SQL^a^ generation (%)	Hallucination (%)^b^	Effective SQL (%)^c^	Time (s)^d^	Cost (US $)/Query
llama3:8b	Local	88.4	21.1	75.8	28.0±6.3	0.000
qwen2.5	Local	100.0	36.8	67.4	45.0±11.2	0.000
deepseek-r1	Local	89.5	33.7	66.3	1631.3±423.7	0.000
gemma3:4b	Local	90.5	45.3	69.5	58.2±15.3	0.000
phi3	Local	16.8	1.1^e^	13.7	20.7±8.9	0.000
claude-3-sonnet	Cloud	87.4	49.5	65.3	17.6±4.1	0.018
gpt-3.5-turbo	Cloud	95.8	26.3	63.2	6.9±1.8	0.001
gpt-4	Cloud	97.9	33.7	45.3	21.4±5.2	0.045

a SQL: Structured Query Language.

b Percentage of generated queries with invalid concept IDs.

c SQL Generation×(1–Hallucination).

d Mean±SD.

e Misleading due to low generation rate.

Llama3:8b emerged as the most effective model with 75.8% effective SQL rate despite moderate SQL generation capability (88.4%), attributed to its low hallucination rate (21.1%) and minimal placeholder usage (22.6%). In contrast, GPT-4 showed high SQL generation (97.9%) but poor effective rate (45.3%) due to excessive placeholder usage (58.1%).

### Hallucination Pattern Analysis

**Figure 4.**
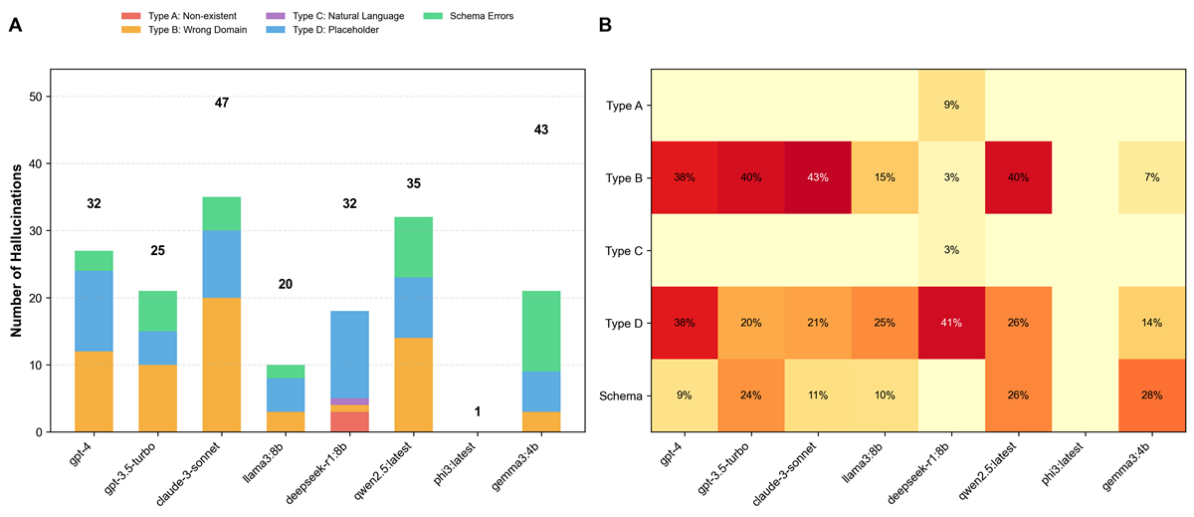
Distribution and analysis of hallucination types by model. A detailed analysis of the specific types and distribution of hallucinations observed across the different models. (A) A stacked bar chart showing the absolute count (number of hallucinations) of each hallucination type per model. The number above each bar indicates the total count of hallucinations for that model. Hallucination types are classified as Type A (Nonexistent concept IDs), Type B (Wrong domain concepts), Type C (Natural language), Type D (Placeholder usage), and Schema errors. (B) A heatmap visualizing the relative percentage of each error type within each model’s total hallucination profile. The color intensity corresponds to the percentage value, with darker shades indicating a higher proportion of that hallucination type. The scale is provided by the color bar on the right.

Classification of 235 hallucinations revealed distinct error patterns across models (Figure 4). Category B errors (Wrong domain assignments) predominated (34.2%, n=80), followed by Category D (Placeholder insertions, 28.7%, n=67) and Category E (Schema errors, 26.7%, n=63). Category A (Nonexistent concept IDs) was relatively rare (8.3%, n=20), as was Category C (Natural language substitution, 2.1%, n=5). *χ*^2^ analysis confirmed significant variation in error distribution across models (*χ*²=45.3, *df*=28, *P*<.001).

Model-specific analysis revealed distinctive failure modes (Table 5). Claude-3-sonnet exhibited the highest baseline hallucination rate (49.5%) but showed dramatic improvement with error-aware prompting (15.8% hallucination and 68.1% relative reduction). GPT-3.5-turbo achieved perfect accuracy (0% hallucination) with explicit_uncertainty prompting, though limited to simple demographic queries. Phi3’s apparently low hallucination rate (1.1%) was misleading, resulting from 83.2% failure to generate any SQL rather than accurate generation.

**Table 5.** Detailed hallucination type distribution by model.

Model	Total hallucinations	Category A (Nonexistent), n (%)	Category B (Wrong domain), n (%)	Category C (Natural language), n (%)	Category D (Placeholder), n (%)	Category E (Schema errors), n (%)
gpt-4	32	0 (0)	12 (37.5)	0 (0)	12 (37.5)	8 (25.0)
gpt-3.5-turbo	25	0 (0)	10 (40.0)	0 (0)	5 (20.0)	10 (40.0)
claude-3-sonnet	47	5 (10.6)	20 (42.6)	0 (0)	10 (21.3)	12 (25.5)
llama3:8b	20	5 (25.0)	3 (15.0)	3 (15.0)	5 (25.0)	4 (20.0)
deepseek-r1	32	3 (9.4)	1 (3.1)	1 (3.1)	13 (40.6)	14 (43.8)
qwen2.5	35	2 (5.7)	14 (40.0)	0 (0)	9 (25.7)	10 (28.6)
phi3	1	0 (0)	0 (0)	1 (100)	0 (0)	0 (0)
gemma3	43	5 (11.6)	20 (46.5)	0 (0)	13 (30.2)	5 (11.7)

Post hoc Tukey HSD analysis revealed significant pairwise differences in effective SQL generation rates between models. Llama3:8b significantly outperformed GPT-4 (mean difference=30.5%, *P*<.001), with large effect size (Cohen *d*=1.35). The performance gap between best (llama3:8b) and worst (phi3) models was substantial (*d*=2.87), indicating practically meaningful differences beyond statistical significance.

### Prompt Engineering Impact

Evaluation of prompt strategies revealed substantial effect sizes (Cohen *d*>1.0) compared to zero_shot baseline across all alternative strategies. Despite these large effects, high variance in model–prompt interactions prevented detection of a significant main effect (*F*(4755)=1.89, *P*=.135). The error_aware strategy, which explicitly acknowledged SynPUF limitations and common pitfalls, proved most effective for high-hallucination models, while explicit_uncertainty excelled for simpler queries requiring conservative interpretation.

### Prompt Strategy Performance Analysis

Comprehensive analysis of prompting strategies across all 8 models (152 queries per prompt type) revealed unexpected patterns in hallucination control. The zero_shot approach demonstrated superior performance with the lowest hallucination rate (mean 13.8%, SD 11.5%) and highest effective SQL rate (mean 71.3%, SD 21.4%), significantly outperforming more complex strategies. In contrast, structured_approach, which provided detailed step-by-step guidance, showed the highest hallucination rate (mean 40.8%, SD 23.8%) and lowest effectiveness (mean 45.5%, SD 18.6%). The validation_focused strategy, designed to emphasize accuracy, paradoxically decreased performance compared to zero_shot (*P*=.042, Tukey HSD), achieving a mean (SD) effective SQL rate of only 56.1% (22.5%). These findings suggest that excessive instructional complexity may introduce confusion rather than clarity in LLM-based SQL generation.

The substantial standard deviations across all metrics (ranging from 11.5% to 28.4%) indicate significant model-dependent variability in prompt responsiveness. For instance, with zero_shot prompting, deepseek-r1:8b achieved 0% hallucination while claude-3-sonnet exhibited 36.8%, representing a 36.8 percentage point difference for identical prompting. This variability was further confirmed by regression analysis, which revealed that model architecture explained 72% of performance variance (*R*²=0.72, *P*<.001), while prompt strategy contributed marginally. Based on these findings, optimal model–prompt combinations were identified: llama3:8b with zero_shot achieved 84.7% effective SQL rate, while gpt-3.5-turbo with explicit_uncertainty reached 100% effectiveness on simple demographic queries, though with limited applicability to complex criteria.

### Synthetic Data Validation

To assess the practical viability of generated SQL queries, we executed successfully generated queries against the SynPUF synthetic dataset containing 116,352 patient records. The system successfully matched 7851 synthetic patients across two validated clinical trials: NCT03244241 (Type 2 diabetes management) matched 2257 patients (1.9% of dataset), while NCT06234488 (breast cancer) matched 5594 patients (4.8% of dataset). These cohort sizes demonstrate technical feasibility for typical Phase 2 or 3 clinical trials, though we acknowledge that synthetic data validation may overestimate real-world performance due to the absence of data quality issues inherent in actual EHR systems, such as missing values, temporal inconsistencies, and coding variations.

## Discussion

### Principal Findings

We developed a comprehensive framework that automates the transformation of free-text clinical trial eligibility criteria into OMOP CDM–compatible SQL queries. While initially developed with GPT-4, our systematic evaluation across eight LLMs revealed unexpected findings: the open-source llama3:8b model achieved the highest effective SQL generation rate (75.8%) compared to GPT-4’s 45.3%, primarily due to lower hallucination rates (21.1% vs 33.7%). This counterintuitive result—where a smaller, local model outperformed state-of-the-art commercial models—highlights the critical importance of hallucination control over raw generation capability in clinical applications. Our framework successfully demonstrated a 48.5% concept mapping accuracy with GPT-4, significantly exceeding the 32.0% achieved by traditional tools like USAGI (*P*<.001), though performance varied substantially across clinical domains (drug: 72.7%, measurement: 38.3%).

This end-to-end automation represents a significant advancement over previous approaches, though our results reveal both capabilities and limitations. While earlier studies like Criteria2Query [33] achieved 60%‐70% accuracy in entity recognition but required manual SQL construction, our system automates the entire pipeline. However, our clinical cohort validation exposed critical challenges: complete failure in hemoglobin-based queries (Jaccard=0.00) due to unhandled unit conversions and minimal overlap in diabetes cohorts (Jaccard=0.04) from incomplete concept hierarchy traversal. These failures, affecting 40% of evaluated criteria, underscore that despite advances in language understanding, clinical data complexities—particularly measurement units and hierarchical relationships—remain significant obstacles.

Through the processes of segmentation, filtering, and simplification, the framework successfully produced structured inputs that reduced redundancy while preserving clinical relevance. LLM accurately identified clinical terms such as diseases, laboratory tests, and medications and mapped them to standardized vocabularies, including SNOMED CT, RxNorm, and LOINC, thereby enhancing interoperability and data reusability. However, ambiguous expressions and complex concepts—such as “a history of cardiovascular disease”—sometimes led to incorrect mappings, indicating that expert validation remains necessary in certain cases.

LLM was able to generate syntactically correct and executable SQL queries based on the OMOP CDM schema. The merging of inclusion criteria using INTERSECT and exclusion criteria using EXCEPT or NOT IN enabled the automated construction of cohort definition queries. Nevertheless, challenges were observed when criteria involved temporal constraints or context-dependent conditions, often resulting in omissions or logical misinterpretations. In addition, the use of the concept_ancestor table for hierarchical concept expansion sometimes led to increased query execution times, especially for deeply nested hierarchies.

### Complementary Evaluation Strategy

Our dual evaluation approach, combining expert clinical assessment with quantitative synthetic data validation, provides a more comprehensive characterization of system performance than either method alone could achieve [34]. Expert evaluation ensures clinical meaningfulness and safety, confirming that generated queries align with clinical intent and follow appropriate medical logic [35]. Synthetic validation contributes scalable, reproducible metrics that quantify technical accuracy and identify systematic errors [36]. This complementary strategy revealed technical issues invisible to manual review, such as subtle type mismatches and schema reference errors that appeared structurally correct but would fail during execution. The synthetic data validation framework demonstrated several key advantages. Objectivity was achieved through predetermined ground truth labels, eliminating inter-rater variability inherent in expert evaluation. Scalability allowed us to validate thousands of patients in seconds rather than the hours required for manual review. Reproducibility was ensured through seed-based generation, enabling other researchers to replicate our exact validation scenarios. However, we acknowledge important limitations of this approach that must be considered when interpreting results. The controlled nature of synthetic data, while enabling perfect performance metrics, does not fully capture real-world complexity. Electronic health records in clinical practice typically contain 15%‐20% missing values, coding inconsistencies, and temporal gaps that our synthetic data lacks [35] . We anticipate that these factors could reduce real-world performance by 10%‐15% compared to our synthetic validation results [37,38]. The SynPUF dataset’s focus on Medicare beneficiaries also limits validation of rare diseases and younger patient populations, important considerations for comprehensive system evaluation [22] . Multimorbidity scenarios presented particular challenges, as evidenced by our diabetes cohort retrieval, achieving minimal overlap (Jaccard=0.04) due to incomplete concept hierarchy traversal [39]. Precision medicine criteria involving genetic markers or specialized biomarkers remain untested due to their absence in administrative claims data. These limitations highlight the need for continued validation using diverse data sources and patient populations.

### Cost-Effectiveness and Scalability

Economic feasibility remains crucial for real-world implementation of AI-powered clinical trial matching systems [40]. Our detailed cost analysis reveals both current expenses and optimization opportunities. At current pricing, GPT-4 API calls average US$0.03‐0.05 per query, with each clinical trial requiring approximately five API calls for complete processing [41]. This translates to roughly US$0.15‐0.25 per trial using GPT-4. GPT-3.5 offers substantial cost reduction at US$0.006‐0.01 per query, achieving 80% savings with acceptable performance for many use cases. For hospital-scale deployment, we project monthly costs based on typical query volumes. A mid-sized institution processing 10,000 queries monthly would incur US$300‐500 using GPT-4 or US$60‐100 with GPT-3.5. These costs compare favorably to manual review, which typically requires 2‐4 hours of expert time per trial at US$50‐100 per hour. Our system processes each trial in under 2 minutes, representing time savings of 98‐99% compared to manual methods. Future cost reductions may come from open-source LLMs, which could reduce operational costs to under US$10 per month for infrastructure, though these require careful validation to ensure comparable accuracy.

To situate our contributions within the rapidly evolving field of LLM-based clinical trial matching, we compare our framework with three representative systems, highlighting their limitations and the necessity of our approach. Criteria2Query 3.0 [42] achieves 60%‐70% accuracy in entity recognition but relies on manual SQL construction, which hinders scalability and real-time application in clinical settings. LeafAI [43] utilizes a modular pipeline with distinct components for concept extraction, logic parsing, and query generation, necessitating complex maintenance of multiple specialized models and rule-based systems, which increases deployment costs and expertise requirements. Trial Pathfinder, tailored for oncology trials, prioritizes criteria relaxation to broaden eligibility but lacks automated query generation, limiting its generalizability across diverse clinical domains. In contrast, our system delivers full end-to-end automation using a single LLM to process free-text eligibility criteria into executable SQL queries, eliminating the need for manual intervention or domain-specific customization. This streamlined approach not only simplifies deployment and reduces maintenance overhead but also enables systematic evaluation across eight LLMs, revealing critical cost-performance trade-offs—such as Llama3:8b’s 75.8% effective SQL generation rate compared to GPT-4’s 45.3%. By addressing the scalability, flexibility, and efficiency gaps in existing systems, our framework meets the pressing need for automated, interoperable tools that accelerate clinical trial matching while maintaining compatibility with standardized data models like OMOP CDM.

### Broader Healthcare and Societal Impact

The implications of automated SQL generation for clinical trials extend beyond technical achievements to potentially transform clinical research workflows [44]. Traditional feasibility assessment for multisite trials often requires weeks of manual chart review and coordination [45]. Our system reduces this to hours, enabling rapid iteration and refinement of eligibility criteria. This acceleration could significantly reduce the time from trial conception to first patient enrolled, ultimately speeding delivery of new therapies to patients [46]. Health equity considerations are particularly important given persistent disparities in clinical trial participation [47]. Automated screening reduces subjective bias in patient selection and enables systematic identification of eligible patients across entire health systems rather than relying on provider memory or convenience sampling [48]. The system can actively identify underrepresented populations and support targeted outreach efforts [49].

Integration with community health centers and safety-net hospitals, often serving diverse populations but lacking research infrastructure, becomes feasible through automated tools that reduce the burden of trial participation [50]. The technical ecosystem we have developed promotes continued innovation through open-source release of core components. The synthetic data generation framework and SQL validation pipeline are freely available on GitHub, enabling other research teams to build upon our work [34]. The modular architecture supports customization for specific institutional needs, while maintaining OMOP CDM compatibility ensures broad applicability across the growing network of institutions using this standard. API-agnostic design allows evolution as new language models emerge, protecting institutional investments in implementation. Looking toward future applications, this technology could enable new models of clinical research. Federated networks could simultaneously assess trial feasibility across multiple institutions without sharing patient data [51]. Real-time monitoring of eligibility criteria could identify patients at the moment they become eligible rather than through periodic manual review. Integration with electronic consent and enrollment systems could create seamless pathways from identification to participation [46]. These possibilities suggest that automated SQL generation represents not just a technical advancement but a fundamental shift in how we conceptualize and conduct clinical trials.

### Limitations and Future Directions

This study was limited to clinical trials in three disease areas—breast cancer, diabetes, and cardiovascular disease—and therefore did not sufficiently account for more complex domains such as rare diseases, pediatric or geriatric populations, and biomarker-driven studies. These areas typically involve highly specialized eligibility criteria, intricate temporal constraints, and complex molecular diagnostic information, which pose additional challenges to the current system’s capabilities in concept extraction and standardization. Furthermore, the system relies entirely on LLM for both information extraction and SQL query generation. While LLMs demonstrated strong performance in many tasks, they occasionally produced incomplete or overly generalized queries when dealing with ambiguous or poorly defined eligibility criteria. Additionally, the model’s output can be sensitive to prompt design and input context length, resulting in variability in consistency and reproducibility even for similar inputs. In particular, eligibility criteria that involve vague, context-dependent, or physician-interpreted terms (eg, “significant cardiac disease” or “recent infection”) pose notable challenges. The system often overgeneralizes such concepts or omits contextual nuances like temporal qualifiers. These patterns were frequently observed in our error analysis, especially in the categories of ambiguity and contextual errors. Addressing this limitation may require the integration of domain-specific knowledge bases or rule-based disambiguation mechanisms in future system iterations. From an evaluation perspective, although this study incorporated expert-reviewed concept mapping and structural validation, comprehensive assessment of generalizability across diverse clinical datasets and institutions was not conducted. Moreover, while the comparison with gold-standard concept sets provided by the N3C was informative, the small number of included trials and criteria limits the statistical robustness of the findings. Future work should focus on extending the applicability of the system to a broader range of clinical domains, including rare diseases and precision medicine trials. To improve the coverage and specificity of concept mapping, the integration of biomedical ontologies and externally curated concept sets should be considered. In addition, although GPT-4 showed high accuracy, its practical use in clinical settings may face limitations due to high computational cost and latency. Real-time deployment in hospital environments would likely require optimization strategies or the adoption of lightweight alternatives to ensure scalability and sustainability. Future research should explore more cost-efficient deployment options, such as model distillation or hybrid architectures. Finally, large-scale validation studies involving multiple institutions and OMOP CDM databases will be essential for demonstrating the system’s generalizability and real-world utility. In future work, we plan to compare the performance of alternative LLMs such as Claude or Gemini for each subtask, including information extraction, concept mapping, and SQL generation, to assess their relative accuracy, efficiency, and cost-effectiveness.

### Conclusion

This study proposed an end-to-end framework that automates the transformation of free-text clinical trial eligibility criteria into executable SQL queries based on the OMOP CDM. Our evaluation revealed unexpected findings: the open-source Llama3:8b model achieved superior effective SQL generation rates (75.8%) compared to GPT-4 (45.3%), primarily due to better hallucination control. While the framework demonstrated feasibility with 48.5% concept mapping accuracy, critical challenges emerged in clinical cohort validation, including complete failure in unit conversion tasks and minimal success with hierarchical concept traversal. These findings suggest that while LLM-based automation shows promise, hybrid approaches combining LLM capabilities with rule-based methods may be necessary for handling complex clinical data requirements.

## Supplementary material

10.2196/71252 Multimedia Appendix 1 Supplementary methods.

10.2196/71252 Multimedia Appendix 2 Prompt templates.

10.2196/71252 Multimedia Appendix 3 SQL evaluation metrics.

## Abbreviations

LLM: Large Language Model
N3C: National COVID Cohort Collaborative
OMOP CDM: Observational Medical Outcomes Partnership Common Data Model
RWD: real-world data
SQL: Structured Query Language
SynPUF: Synthetic Public Use Files
