# Supplementary material for: Large Language Models for Automating Clinical Trial Criteria Conversion to Observational Medical Outcomes Partnership Common Data Model Queries: Validation and Evaluation Study
Source: JMIR Med Inform. 2025 Oct 16;13:e71252. doi: 10.2196/71252 (PMC12530336; doi:10.2196/71252)
Supplement: Multimedia Appendix 1 [file medinform-v13-e71252-s001.docx]

**Supplementary Methods**

This appendix provides a detailed description of the internal architecture and processing steps of the automated system designed to transform clinical trial eligibility criteria into OMOP CDM-compliant SQL queries. The process consists of six sequential modules, as illustrated in Figure S1: (1) preprocessing, (2) information extraction and standard code mapping, (3) SQL query generation, (4) large language model (LLM)-based evaluation, (5) SQL merging, and (6) execution. Each module progressively transforms unstructured free-text into structured, executable queries for identifying eligible patient cohorts.

**Overview of the End-to-End System**


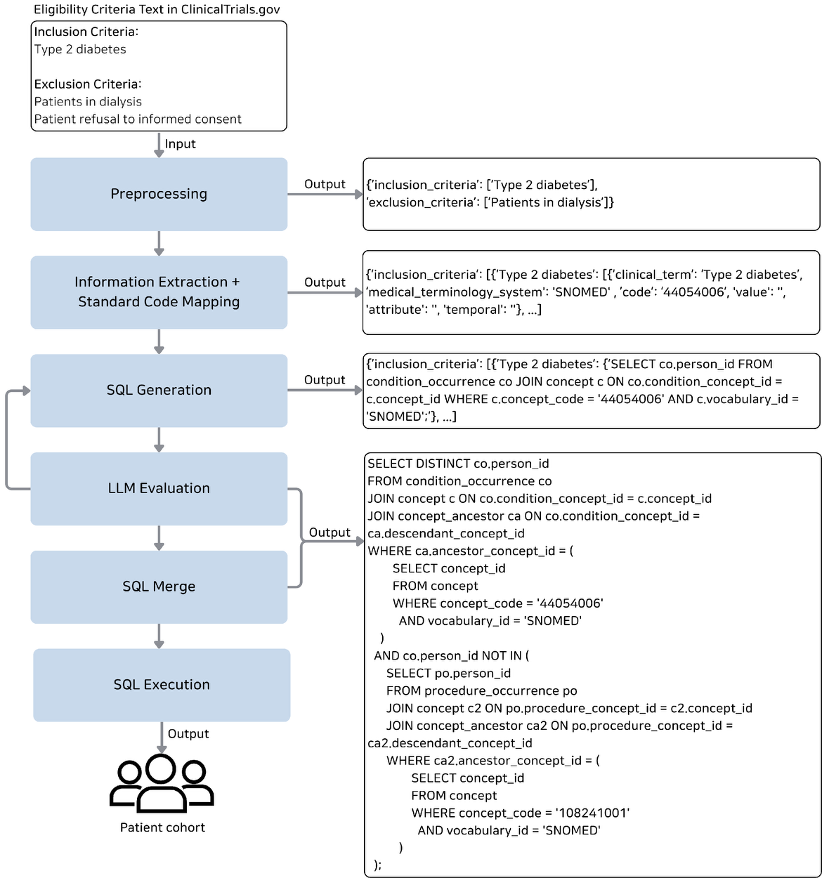


**Figure S1. End-to-end pipeline for automated conversion of clinical trial eligibility criteria to OMOP CDM SQL queries.** The system transforms free-text criteria through six sequential modules: preprocessing, information extraction with standard code mapping, SQL generation, LLM-based evaluation, SQL merging, and execution, ultimately identifying eligible patient cohorts. Each module shows example outputs demonstrating the progressive transformation from unstructured text to structured queries. Input example shows inclusion criteria for Type 2 diabetes, exclusion criteria for patients in dialysis and patient refusal to informed consent. The preprocessing module filters trial-specific criteria and outputs structured clinical criteria. Information extraction identifies clinical terms with SNOMED codes and attributes. SQL generation creates OMOP-compliant queries with concept hierarchies. LLM evaluation refines queries for accuracy. SQL merge combines inclusion and exclusion logic. Finally, SQL execution retrieves the eligible patient cohort.

**Step1. Preprocessing**

The preprocessing module applies a structured, rule-based framework to normalize and simplify raw eligibility criteria. This module consists of three stages: segmentation, filtering, and simplification.

In the segmentation stage, each eligibility criterion is divided into inclusion and exclusion categories and further decomposed into individual logical units. Logical operators such as “AND” and “OR,” as well as structural punctuation like colons, are preserved to maintain the logical structure and hierarchy within the criteria.

In the filtering stage, trial-specific criteria that are irrelevant to clinical databases—such as those related to informed consent, geographic location, or study logistics—are excluded. This filtering is performed in two phases. First, domain experts (a clinician and a medical informaticist) manually classified the criteria into queryable and non-queryable groups using a predefined taxonomy. Then, a GPT-4-based classifier was employed to automatically assign each criterion to one of ten predefined categories, filtering out those marked as non-queryable.

In the simplification stage, the system removes content that is linguistically valid but not interpretable in a structured query context. For example, ambiguous phrases, subjective language, or vague measurements without thresholds are removed. This stage was guided by simplification rules adapted from Fang et al.'s participatory design study on the ARCADIA trial [1]. The retained content includes only elements that can be reliably translated into OMOP CDM queries. Detailed rules applied during this process are summarized in Table S1.

**Table S1**. Simplification Rules

| **Level** | **Rule** |
| --- | --- |
| Sentence-level | Remove statements about optional or physician-discretionary procedures. |
|  | Remove criteria requiring patient interview or judgment by investigators. |
|  | Remove informed consent or willingness statements. |
| Phrase-level | Always retain demographic and pregnancy-related terms. |
|  | Remove imaging modality references unless they include clinical values. |
|  | Remove vague group labels, retain specific examples. |
|  | Remove measurement phrases that lack explicit thresholds or negation. |
|  | Remove abbreviations if the full term is provided. |

**Step2: Information Extraction and Concept Mapping**

To convert clinical trial eligibility criteria into OMOP CDM-compliant SQL queries, our system performs two essential processes: Information Extraction and Standard Concept Mapping. These steps enable the transformation of free-text criteria into a structured, semantically standardized representation that aligns with OMOP vocabulary specifications and supports automated query generation.

**Information Extraction from Criteria**

The first step involves decomposing free-text eligibility criteria into five core elements that reflect the logical and clinical structure of each statement:

- Clinical Term: The core medical concept mentioned in the criteria, such as a disease, symptom, procedure, or therapeutic intervention (e.g., “diabetes mellitus,” “chemotherapy”).

- Value: Numerical or categorical values associated with the clinical term, typically representing threshold-based inclusion or exclusion (e.g., “HbA1c ≥ 7%,” “positive result”).

- Attribute: Qualifiers or modifiers that refine the interpretation of the clinical term, such as comparative operators (e.g., “greater than,” “equal to”) or range indicators (e.g., “at least,” “no more than”).

- Temporal: Time-related constraints indicating when the condition or event must occur (e.g., “within the past 6 months,” “during hospitalization”).

- Negation: Indicators of the explicit absence of a condition or event, critical for identifying exclusion criteria (e.g., “no prior history of stroke”).

These elements are explicitly parsed from the original text through linguistic and semantic analysis and form the basis for concept normalization and SQL generation.

**Mapping to Standard Medical Terminologies**

After extracting the relevant elements, each Clinical Term is mapped to standardized medical vocabularies to ensure consistency and interoperability with OMOP CDM conventions. This mapping includes the following components:

- Medical Terminology System: The extracted clinical concept is aligned with a standard vocabulary such as SNOMED CT, ICD-10, RxNorm, or LOINC, depending on its semantic type.

- Code: Each concept is assigned a unique identifier (e.g., SNOMED concept ID) within its respective terminology system, allowing for unambiguous referencing across datasets.

- Domain Classification: Based on contextual and semantic cues, each concept is classified into one of OMOP’s predefined domains: Condition, Drug, Measurement, Procedure, Device, or Observation.

To address ambiguity between domains (especially common among terms that could be interpreted as either conditions or measurements), we embedded explicit domain classification rules into the GPT-4 prompting framework. For example:

- Diagnostic concepts such as “diabetes mellitus” are classified as Condition.

- Quantitative criteria like “blood pressure > 140 mmHg” are categorized as Measurement.

- Subjective complaints such as “complains of fatigue” fall under the Observation domain.

This two-tiered structure—separating linguistic extraction from semantic normalization—ensures high fidelity to the original clinical intent while achieving interoperability with OMOP CDM standards. It also supports scalable, high-accuracy SQL generation for downstream clinical data analysis and cohort identification.

**Step3: SQL Query Generation**

The system then generates OMOP CDM-compatible SQL queries through a three-stage process: query generation, LLM-based evaluation, and query merging.

In the query generation phase, SQL statements are constructed based on the extracted elements and mapped codes. The OMOP CDM’s concept_ancestor table is leveraged to include descendant concepts of broader clinical terms, ensuring comprehensive coverage. For example, a high-level term like “chemotherapy” will automatically encompass specific drugs or procedures classified under it.

Next, each generated SQL query is passed back to GPT-4 for validation. The model reviews the syntax, logic, and schema alignment, and revises any errors or inefficiencies. When modifications are made, GPT-4 also provides a natural language explanation describing the rationale behind the revision—for instance, incorrect column use, missing joins, or domain mismatches.

Finally, the system merges multiple individual queries into a single cohort-defining SQL statement. Inclusion criteria are combined using the INTERSECT operator to retain only patients who satisfy all required conditions. Exclusion criteria are combined using UNION and applied using NOT IN or EXCEPT clauses to remove ineligible patients from the final cohort. This merging strategy ensures accurate population definition in a reproducible and scalable manner.

**References**

1. Fang Y, Kim JH, Idnay BR, et al. Participatory Design of a Clinical Trial Eligibility Criteria Simplification Method. Stud Health Technol Inform. 2021;281:984-988. doi:10.3233/SHTI210325
