# Supplementary material for: Large Language Models for Automating Clinical Trial Criteria Conversion to Observational Medical Outcomes Partnership Common Data Model Queries: Validation and Evaluation Study
Source: JMIR Med Inform. 2025 Oct 16;13:e71252. doi: 10.2196/71252 (PMC12530336; doi:10.2196/71252)
Supplement: Multimedia Appendix 2 [file medinform-v13-e71252-s002.docx]

**Table S1.** Examples of Prompts

| **Task** | **Prompt** |
| --- | --- |
| Segmentation | Your task is to segment the eligibility criteria text into individual criteria.  ## Segmentation rule  - Group logically connected items ('or', 'and', etc.)  - Include sub-criteria or examples in the same criterion.  {format_instructions}  Eligibility criteria text: {criteria_text} |
| Classification | Your task is to classify the given criterion into one of the following categories.  ## Classification categories  - Demographic Eligibility: Related to basic demographic characteristics like age, gender, race, and ethnicity.  - Location and Accessibility: Focuses on geographic location and accessibility to study sites.  - Consent and Comprehension: Ensures participants can give informed consent.  - Study Commitment: Assesses participants' willingness and ability to comply with the study procedures.  - Health and Medical History: Pertains to participants' past and present health conditions.  - Behavioral and Lifestyle: Concerns participants' lifestyle choices and behaviors.  - Treatment and Medication History: Involves history of treatments and medication use.  - Disease or Condition Specific: Relates directly to the disease or condition being studied.  - Participant Relationship: Includes criteria related to social and familial relationships.  - Regulatory and Ethical: Ensures the study meets regulatory requirements and ethical standards.  {format_instructions}  Criterion: {criterion}  Output: |
| Simplification | ##Task  - Remove unnecessary or irrelevant text before converting eligibility criteria into SQL queries based on the OMOP CDM.  - Simplify the given criterion based on the following ##Sentence-level Rules and ##Phrase-level Rules.  ##Sentence-level Rules  - Information related to research activity authorization should be omitted.  - Criteria requiring patient interview or investigator judgment should be omitted.  - Criteria related to informed consent or willingness to comply with the study protocol should be omitted.  ## Phrase-level  - Phrases related to demographics (e.g., age and gender) and pregnancy status should always be retained.  - Phrases referring to imaging procedures for ruling out conditions should be omitted unless they include clinical findings.  - Phrases containing broad or vague clinical concepts should be omitted, while specific and clearly defined examples should be retained.  - Phrases combining general disease conditions with specific measurements should be simplified by retaining only the measurements.  - Phrases referring to measurements without explicit or implicit value thresholds should be omitted.  {format_instructions}  Criterion: {criterion}  Output: |
| Information Extraction & Concept Mapping | ## Task:  - Extract and structure clinical terms from simplified clinical trial eligibility criteria for SQL query generation.  - Each criterion description can include multiple clinical terms.  - The processing pipeline must follow these steps:  1. Extract clinical terms from the criteria.  2. For each clinical term, identify the associated value, attribute (e.g., comparison operators), temporal information, and negation, if present.  3. Map each clinical term to a standardized concept by referencing the OHDSI Athena database (https://athena.ohdsi.org/search-terms/start) to obtain the appropriate medical terminology system, code, and domain.  - If a clinical term appears in both abbreviation and full name form, retain only the full name.  - If a clinical term is provided only as an abbreviation, expand it to its full name prior to processing.  - Your output must follow the definitions in ##Term Definitions and the rules in ##Domain Classification Rules.  ## Term Definitions:  - clinical_term: The medical concept or condition mentioned in the criteria (e.g., "diabetes mellitus").  - value: A numeric or categorical value associated with the clinical term.  - attribute: A comparison operator that qualifies the value (e.g., "greater than").  - temporal: A time-related condition (e.g., "within the past 6 months").  - negation: Indicates whether the clinical concept is explicitly excluded.  - medical_terminology_system: The standardized terminology used to map the clinical term (e.g., SNOMED CT, LOINC).  - code: The standardized concept code for the term.  - domain: The OMOP CDM domain to which the term belongs (e.g., Condition, Measurement, Drug).  ## Domain Classification Rules:  - Use "Condition" for diseases or diagnoses.  - Use "Measurement" for quantifiable observations. (e.g., “blood pressure > 140 mmHg”)  - Use "Observation" for subjective symptoms. (e.g., “complains of fatigue”)  - Use other OMOP domains (Drug, Procedure, etc.) as appropriate.  - When in doubt, prefer "Measurement" if numeric thresholds or units are present.  {format_instructions}  Simplified criterion (example1):  {info_criteria_example1}  Information extraction (example1):  {info_example1}  Simplified criterion (example2):  {info_criteria_example2}  Information extraction (example2):  {info_example2}  Simplified criterion:  {criterion} |
| SQL Generation | You are a medical data engineer responsible for writing precise SQL queries using the OMOP CDM PostgreSQL schema.  ## Task:  - Use the provided simplified criterion and extracted data fields to write a PostgreSQL SQL query.  - Always use concept_code by joining with the `concept` table. Do not use concept_id directly.  - Choose the appropriate OMOP table (e.g., condition_occurrence, observation, drug_exposure) based on the clinical term and context.  - Apply filters such as "between", "greater than", "within X months", or negations accordingly.  - Return only ‘person_id’ in the final SELECT clause.  ## Note:  - Each data field contains: `clinical_term`, `value`, `attribute`, `temporal`, `negation`, and `standard_code` (list of tuples: `(vocabulary, code)`).  - You may use JOINs on the `concept` table using concept_code (e.g., `JOIN concept c ON t.concept_id = c.concept_id AND c.concept_code = 'XYZ'`).  ##Schema  - person(person_id, gender_concept_id, year_of_birth, month_of_birth, day_of_birth, race_concept_id, ethnicity_concept_id)  - condition_occurrence(condition_concept_id, condition_start_date, condition_end_date, condition_type_concept_id)  - observation(observation_id, person_id, observation_concept_id, observation_date, value_as_number, value_as_string)  - procedure_occurrence(procedure_occurrence_id, person_id, procedure_concept_id, procedure_date)  - drug_exposure(drug_exposure_id, person_id, drug_concept_id, drug_exposure_start_date, drug_exposure_end_date, quantity, days_supply, route_concept_id, dose_unit_concept_ id)  - device_exposure(device_exposure_id, person_id, device_exposure_start_date, device_exposure_end_date, device_type_concept_id, quantity, device_source_ concept_id)  - concept(concept_id, concept_code, concept_name, domain_id, vocabulary_id, concept_class_id, standard_concept)  {format_instructions}  Output JSON only.  Simplified criterion(example1):  {sql_generation_criterion_example1}  Data fields(example1):  {data_fields_example1}  SQL query:(example1)  {generated_sql_example1}  Simplified criterion(example2):  {sql_generation_criterion_example2}  Data fields(example2):  {data_fields_example2}  SQL query:(example2)  {generated_sql_example2}  Simplified criterion:  {criterion}  Data fields:  {data_fields}  SQL query: |
| LLM-based Evaluation | You are a clinical informatics and OMOP-CDM expert.  You specialize in verifying whether SQL queries accurately implement clinical eligibility criteria using OMOP-CDM conventions and best practices.  ## Task  - If the SQL is correct, return it unchanged and specify "is_modified": "No".  - If the SQL needs improvement or correction, return the updated SQL, explain the reason, and set "is_modified": "Yes".  ## Instructions  - The SQL must use standard OMOP-CDM tables such as condition_occurrence, measurement, observation, drug_exposure, procedure_occurrence, visit_occurrence, person, and concept.  - When referencing clinical concepts:  - Ensure that the concept table is properly joined using concept_id.  - Use concept_code and vocabulary_id to identify the correct clinical term.  - If a parent concept is referenced, descendant concepts must be included using the concept_ancestor table.  - All date-based conditions (e.g., condition_start_date, measurement_date) must fall within the person’s observation_period.  - For measurement values:  - value_as_number must be properly constrained.  - unit_concept_id should be checked for correctness or consistency.  - For negation or absence logic, use NOT EXISTS or LEFT JOIN ... IS NULL as appropriate.  - Use INTERSECT to combine inclusion criteria (AND logic), UNION to combine multiple exclusion criteria (OR logic), and EXCEPT or NOT IN to exclude patients meeting exclusion criteria.  - The SELECT clause must only return DISTINCT person_id. No other fields are allowed.  - If the SQL correctly implements the criterion, return the original SQL without changes and mark "is_modified": "No".  - If the SQL requires improvement or correction, return the corrected version and provide a clear explanation in the "reason" field, along with "is_modified": "Yes".  - Always include all required fields in the output: simplified_criterion, original_sql, corrected_sql, reason, and is_modified.  - Do not leave any field empty. For example, if no correction is needed, still provide a reason such as "SQL already correct.".  - Only return the JSON object as output.  {format_instructions}  Criterion (example1): {evaluation_criterion_example1}  Original SQL (example1): {original_sql_example1}  Corrected SQL (example1): {corrected_sql_example1}  Criterion (example2): {evaluation_criterion_example2}  Original SQL (example2): {original_sql_example2}  Corrected SQL (example2): {corrected_sql_example2}  Criterion: {criterion}  Original SQL: {original_sql}  Corrected SQL: |
| SQL Merge | You are a clinical informatics expert specializing in OMOP-CDM-based patient cohort selection using SQL.  ## Task  - Generate a single, correct, and optimized SQL query that implements all of the inclusion and exclusion criteria provided below. Each criterion is paired with its SQL implementation.  ## Instructions  - Use `INTERSECT` to combine inclusion criteria (AND logic).  - Use `NOT IN`, `NOT EXISTS`, or `EXCEPT` to apply exclusion logic (patients who meet exclusion criteria must be removed).  - Always use `SELECT DISTINCT person_id` as the final output.  - Maintain valid SQL syntax across OMOP-CDM tables (e.g., `condition_occurrence`, `person`, `concept`).  - Avoid using `NOT (...)` inside a subquery that is itself wrapped with `NOT IN` — this causes logic inversion and incorrect exclusions.  - If an exclusion query uses `NOT (...)`, assume it is logically inverted and rewrite it as a direct positive condition. Then use `NOT IN (...)` on that positive query.  - If a SQL query appears logically flawed or improperly negated, correct it before merging.  - Add a semicolon at the end of the final query.  - Ensure that exclusion logic works by first selecting the patients who meet the exclusion condition (e.g., a diagnosis), then excluding them. Do not try to "negate the negation".  Inclusion Criteria and SQL:  {inclusion_block}  Exclusion Criteria and SQL:  {exclusion_block}  Final merged SQL: |
