# Supplementary material for: Large Language Models for Automating Clinical Trial Criteria Conversion to Observational Medical Outcomes Partnership Common Data Model Queries: Validation and Evaluation Study
Source: JMIR Med Inform. 2025 Oct 16;13:e71252. doi: 10.2196/71252 (PMC12530336; doi:10.2196/71252)
Supplement: Multimedia Appendix 3 [file medinform-v13-e71252-s003.docx]

**Table S1.** Detailed Scoring Criteria for SQL Query Evaluation Metrics

| **Metrics** | **Score** | **Description** |
| --- | --- | --- |
| SQL syntax adherence | 1 | The query is completely invalid and cannot be executed due to significant syntax errors. |
|  | 2 | The query has multiple syntax errors, making correction difficult or requiring significant rewrites. |
|  | 3 | The query contains minor syntax errors but can be corrected with reasonable effort. |
|  | 4 | The query is free of syntax errors and can be executed without modification. |
| CDM schema compliance | 1 | The query uses incorrect or nonexistent tables/columns, failing to align with the schema. |
|  | 2 | The query largely deviates from schema standards, requiring major changes. |
|  | 3 | The query uses some incorrect tables/columns but can be fixed with moderate adjustments. |
|  | 4 | The query strictly adheres to the OMOP CDM schema. |
| Criteria contextual accuracy | 1 | The query does not align with the context or intent at all. |
|  | 2 | The query reflects the context in a very limited manner, requiring substantial reworking. |
|  | 3 | The query partially reflects the context and can be adjusted with moderate corrections. |
|  | 4 | The query accurately captures the context and eligibility criteria without requiring changes. |
| Concept inclusion accuracy | 1 | No necessary concepts are included, or only irrelevant ones are present. |
|  | 2 | Most necessary concepts are missing, requiring significant additions. |
|  | 3 | Some necessary concepts are missing or extraneous ones are included but can be fixed with effort. |
|  | 4 | All required concepts are included, and unnecessary concepts are absent. |
| Concept id correctness | 1 | No correct concept IDs are included, or IDs are entirely irrelevant. |
|  | 2 | Most concept IDs are incorrect, requiring significant effort to replace. |
|  | 3 | Some concept IDs are incorrect but can be fixed with reasonable effort. |
|  | 4 | All concept IDs are correctly identified and used. |
